# Editorial: An insight into multi-omics analysis of dementia disorders

**DOI:** 10.3389/fgene.2023.1206530

**Published:** 2023-05-16

**Authors:** Prachi Srivastava, Anshul Tiwari, Khurshid Ahmad, Neha Srivastava, Prekshi Garg

**Affiliations:** ^1^ Amity Institute of Biotechnology, Amity University Uttar Pradesh, Lucknow Campus, Uttar Pradesh, India; ^2^ Molecular Physiology and Biophysics, Vanderbilt University, Nashville, TN, United States; ^3^ Department of Medical Biotechnology, Yeungnam University, Gyeongsan, Republic of Korea; ^4^ Excelra, Hyderabad, Telangana, India

**Keywords:** dementia, multi-omics, transcriptomics, proteomics, genomics

Dementia is a debilitating and prevalent disorder affecting millions worldwide (Chern and Golub, 2019). The emergence of multi-omics analysis has led to a better understanding of the underlying disease mechanism and its effective treatment. This advanced approach combines data from multiple sources, such as genomics, transcriptomics, proteomics, and metabolomics, that helps understand the complex biological processes resulting in dementia.

The potential benefits of multi-omics analysis in dementia research are significant. Researchers can develop more targeted and effective therapies by identifying key molecular pathways and biomarkers associated with these disorders. Multi-omics analysis may also help to identify subtypes of dementia disorders, allowing for more personalized treatment approaches. For a better understanding of the complex biological processes involved in dementia, the multi-omics analysis combines data from multiple resources leading to new insights and potential treatments for this devastating disorder (Mavrina et al., 2022).

This Research Topic presents eight manuscripts, consisting of five original research papers, one case report, one review, and one mini-review. The manuscripts cover a diverse range of Research Topic related to recent advances and applications of multi-omics analysis in the field of dementia and neurodegenerative disorders. These expert-contributed manuscripts provide valuable insights into the underlying molecular mechanisms of dementia-related disorders as well as new diagnostic and treatment strategies.

Wang et al., examined the upregulated expression of PLAGL2 in gliomas and identified a correlation with negative clinicopathological characteristics, including tumor grade. Moreover, the investigation revealed the role of PLAGL2 as an independent prognostic indicator, not for only progression-free survival (PFS) but also overall survival (OS) in clinical glioma specimens. Therefore, findings highlight the role of PLAGL2 expression in glioma progression and suggest its potential use as a prognostic marker for diagnosis and prognosis. The study’s results could have a significant impact on the diagnosis and treatment of patients with high-grade gliomas.

Alqahtani et al., examined 47 genes associated with dementia and discovered that compositional, selectional, and mutational forces affect codon usage bias (CUB). The study suggested the positive association between high GC content and elevated gene expression levels, hence favoring GC-ending codons over AT-ending codons. The behavior of the TTG codon was unique, indicating the influence of selection on codon usage. The investigation highlights the significance of selection pressure, compositional constraints, and mutational forces in finding the usage of the codon.

Khandia et al., investigated the correlation between gene length and various parameters related to codon usage in genes associated with neurodegenerative disorders. The study suggests the significant correlation of gene length with codon bias and also highlights the preference for GC-ending codons over AT-ending codons. The correlation between codon usage bias and gene length varied depending on the segment size, but gene segments CUB range from 1,2002,000 bp seems to be not affected by gene length. The investigation suggests that the significance of the impact of gene length and codon usage bias can aid in gene expression modulation in cases of defective gene functioning in clinical settings.

Kumari et al., in their study, investigate the changes occurring in gene expression in the temporal cortex of aged control, asymptomatic AD, and symptomatic AD individuals. They identified differentially expressed genes and performed protein-protein interaction network analysis to obtain hub genes in the network. The study highlights the changes in glutamatergic hyperexcitability at early-stage in the AsymAD group and protein synthesis impairment in the symAD group. The findings offer potential therapeutic targets for early intervention in AD.

Khan et al., developed a ligand-based model using machine learning to identify effective AChE inhibitors. They trained and tested the model on experimental data and used it to screen the Maybridge chemical database, leading to the discovery of four highly effective inhibitors with superior binding affinity to a known drug. These compounds have the potential to serve as a foundation for developing anti-AD drugs.

Xue Bin et al., conducted a mini-review that delved into the correlation between BDNF, mental health, and AD. The review mainly focused on the role of nutrition, lifestyle, and environment in BDNF, leading toward the contribution to AD. The review further highlighted the potential new therapeutic targets that could aid in treating neurodegenerative diseases.

A review article by Tiwari and Shukla emphasizes the importance of early diagnosis of AD and the use of the latest technologies such as lipidomics and proteomics for determining the alteration in lipids and proteins level in the biological samples. These techniques could aid in halting the progression of AD and dementia, and the review highlights their potential for early diagnosis and assessment of the disease.

Chen et al., reported a case of Wolfram syndrome 1 (WSF1) gene mutation that initially presented with cognitive impairment and later recurrent cerebral infarction. Brain imaging showed reduced intracranial volume, particularly in the cerebral cortex and cerebellum, and Tau protein deposition without Aβ pathology changes. The findings suggest a possible link between WFS1 and neuronal vulnerability to tau pathology and ischemic damage.

The multi-omics approach extends the unique technology that broadly highlights the characterization of dementia and other neurological diseases at various biological levels. This will help in understanding in-depth disease mechanisms more widely. This approach opened a new viewpoint for the early diagnosis and treatment options for AD patients. This will also help in finding potential targets for better treatment as well as prevention for patients with AD risk or who only have AD symptoms (Badhwar et al., 2020).

Conclusively, the authors and editors of this Research Topic anticipate that the Research Topic of articles will highlight the progress made in the multi-omics analysis and provide a more comprehensive understanding of the complex biological processes involved in dementia, leading to potential new treatments for the disorder. We hope that the articles will motivate, inform, and provide direction to researchers and students in the field.
